# Estimating Consumption to Biomass Ratio in Non-Stationary Harvested Fish Populations

**DOI:** 10.1371/journal.pone.0141538

**Published:** 2015-11-03

**Authors:** Rodrigo Wiff, Ruben H. Roa-Ureta, David L. Borchers, Andrés C. Milessi, Mauricio A. Barrientos

**Affiliations:** 1 Center of Applied Ecology and Sustainability (CAPES), Pontificia Universidad Católica de Chile, Av. Alameda 340, Santiago, Chile; 2 King Fahd University of Petroleum and Minerals, Center for Environment and Water, Dhahran 31261, Saudi Arabia; 3 Centre for Research into Ecological and Environmental Modelling. School of Mathematics and Statistics. University of St. Andrews, The Observatory, Buchanan Gardens, St. Andrews KY16 9LZ, Scotland, United Kingdom; 4 Comisión de Investigaciones Científicas de la Provincia de Bs.As (CIC). Calle 526, 1900, La Plata, Argentina; 5 Instituto Nacional de Investigación y Desarrollo Pesquero (INIDEP), Paseo Victoria Ocampo No. 1, 7600 Mar del Plata, Argentina; 6 Instituto de Matemáticas, Pontificia Universidad Católica de Valparaíso, Blanco Viel 596, Cerro Barón, Valparaíso, Chile; Swedish University of Agricultural Sciences, SWEDEN

## Abstract

The food consumption to biomass ratio (*C*) is one of the most important population parameters in ecosystem modelling because its quantifies the interactions between predator and prey. Existing models for estimating *C* in fish populations are per-recruit cohort models or empirical models, valid only for stationary populations. Moreover, empirical models lack theoretical support. Here we develop a theory and derive a general modelling framework to estimate *C* in fish populations, based on length frequency data and the generalised von Bertalanffy growth function, in which models for stationary populations with a stable-age distributions are special cases. Estimates using our method are compared with estimates from per-recruit cohort models for *C* using simulated harvested fish populations of different lifespans. The models proposed here are also applied to three fish populations that are targets of commercial fisheries in southern Chile. Uncertainty in the estimation of *C* was evaluated using a resampling approach. Simulations showed that stationary and non-stationary population models produce different estimates for *C* and those differences depend on the lifespan, fishing mortality and recruitment variations. Estimates of *C* using the new model exhibited smoother inter-annual variation in comparison with a per-recruit model estimates and they were also smaller than *C* predicted by the empirical equations in all population assessed.

## Introduction

Food consumption of a population is one of the most important quantities required to implement multiespecies models in aquatic ecosystems, because it directly quantifies the intensity of interactions between predator and prey. Regular stock assessment programs provide annual estimates of abundance of the most productive fish stocks of various marine ecosystems around the world. To connect these estimates in multispecies models we need estimates of food consumption to biomass ratio (hereafter *C*) at the population level. This ratio can be seen as the number of times a population eats its own weight during a certain period of time (usually a year), a kind of standardised population consumption rate. Methods for estimating consumption rates of fish at the individual level have been well studied (see [1]). Conversely, estimating *C* at the population level is a laborious and difficult task that is usually done using methods that depend on strong assumptions. This is problematic for most fish species because the strong assumptions of existing methods imply serious limitations. Here, we develop and demonstrate the applicability of a new general method based on data on the population size structure.

Conventional methods to estimate population consumption rates fall roughly into two categories: (*i*) methods, like those of Pauly [2] and Aydin [3], in which experimental and field data are combined to estimate *C* by integrating consumption and biomass over a cohort lifespan, thus providing per-recruit estimator for *C*. (*ii*) Methods that use an empirical relationship between *C* and some environmental and body size attributes [4]. Pauly’s model relies on the assumption of stable age-distribution and the parameters defining individual consumption have no clear biological meaning. Aydin [3] extended Pauly’s model to incorporate biological parameters which describe consumption but this model still relies on the assumption of stable age-distribution. The per-recruit analysis framework used in Pauly [2] and Aydin [3] has two main drawbacks for estimating *C*. First, the use of the specialised von Bertalanffy growth model implies the assumption of an anabolism parameter, *d* = 2/3, but this specific value is unusual for teleost fishes [5]. Second, the assumption of a stable age-distribution may be valid for stationary populations but it may not be useful for fished populations because fishing exploitation often produces inter-annual variations in age-dependent mortality and recruitment [6]. The application of empirical models on the other hand, is straightforward, but these models lack theoretical support, relying on assumptions of constant coefficients across species and environments, and they cannot account for shifts in population structure. These limitations suggest that alternative approaches are needed.

The relationship between the growth rate of an individual fish and the amount of food it ingests has been noted by several authors (e.g. see [7–10]). The existence of this relationship implies that food consumption can be inferred from growth rate [11, 12]. In this reductionist approach, food acquisition is primarily limited by properties of the organism and, consequently, growth rate is a feature of organism design [13, 14]. Thus a number of authors (see e.g., [2, 15]) have proposed models to estimate food consumption from feeding experiment and size-based attributes derived from modelling growth. These models have been widely used to explain processes at the individual level. What is lacking, however, is a quantitative framework that connects individual processes to population attributes. Here we propose such a framework for estimating *C* by modelling individual growth using the generalised von Bertalanffy growth function and incorporating population attributes by using population size structure. This allows changes in *C* due to shifts in population size structure to be modelled.

## Analysis

The von Bertalanffy’s principle states that the rate of growth of an individual is determined by the difference between the build-up of body mass due to energy input and energy expenditures due to maintenance. Growth rate can be described by the following differential equation:

$$\begin{array}{c} \frac{dw}{da}=Hw^{d}-\alpha w^{\eta }\;, \end{array}$$ (1)

where *a* is age and *w* is the body weight. *Hw* ^*d*^ reflects the anabolism term (energy assimilation) and *αw* ^*η*^ reflects the catabolism term (energy loss) and *H* and *α* are proportionality constants for anabolism and catabolism, respectively. In particular, *d* (0 ≤ *d* ≤ 1) and *η* (0 ≤ *η* ≤ 1) are allometric scaling factors for anabolism and energy cost, respectively. When they are set at *d* = 2/3 and *η* = 1 the result is the von Bertalanffy growth function (VBGF) in its original formulation. In a more general setting, *d* can take a value other than 2/3 leading to the generalised VBGF,

$$\begin{array}{c} w\left(a\right)=w_{\infty }{\left(1-\left(1-{\left(\frac{w_{0}}{w_{\infty }}\right)}^{1-d}\right)e^{-ka}\right)}^{\frac{1}{1-d}}\;, \end{array}$$ (2)

where *w*_∞_ is the asymptotic body weight, *w*_0_ is initial body weight, and *k* is the growth coefficient defined as *k* = *α*(1 − *d*). Note here we use the VBGF parameterised in terms of initial *w*_0_ instead of the original formulation which included age at weight zero (*t*_0_). The VBGF parameterised in terms of *w*_0_ makes the growth model more directly interpretable [16].

Temming and Herrmann [17] related the instantaneous consumption rate (*q*) with anabolism using the proportionally constant *A* (0 ≤ *A* ≤ 1) by *q* = (*H*/*A*)*w* ^*d*^. According to Temming [15], *H* can be recast in terms of the generalised VBGF as $H=\frac{k}{\left(1-d\right)w_{\infty }^{\left(d-1\right)}}$.

Thus, an expression for the instantaneous individual consumption rate, in which all parameters have a clear biological meaning is:

$$\begin{array}{c} q\left(w\right)=\frac{k}{A\left(1-d\right)w_{\infty }^{\left(d-1\right)}}w^{d}\;. \end{array}$$ (3)

We proceed as follows to incorporate the fact that the birth dates of individuals in multi-cohort populations are different: Let *w*_t*_ be the body mass of a randomly selected individual in the population at time t* and let *f*_t*_(*w*_t*_) be the probability density function (pdf) of *w*_t*_. We suppose that t* is a pre-determined point in year *y*. For the sake of brevity, we drop the t* and write the pdf of *w* at time t* in year *y* as *f*_*y*_(*w*). The individual consumption rate, *q*(*w*) is then also a random variable and, provided *w* is continuous, the expected value of the population consumption to biomass ratio, *C*(*w*) at time t* in year *y* can be written as:

$$\begin{array}{ccc} E_{f_{y}}\left[C(w)\right] & = & \int_{w_{r}}\;\frac{q(w)}{w}\;f_{y}\left(w\right)\;dw\\ & = & \frac{k}{A\;\left(1-d\right)\;w_{\infty }^{\left(d-1\right)}}\int_{w_{r}}\;w^{\left(d-1\right)}\;f_{y}\left(w\right)\;dw\\ & = & \frac{k}{A\;\left(1-d\right)\;w_{\infty }^{\left(d-1\right)}}\;E_{f_{y}}\left[w^{\left(d-1\right)}\right]\;, \end{array}$$ (4)

where *w*_*r*_ ≔ [*w*_0_, *w*_∞_] and *E* is the expectation operator. Here we assume that the population is composed of individuals that share the same growth parameters (*w*_0_, *w*_∞_, *d*, *k*) and assimilation rate (*A*). If length is treated as a deterministic function of weight, *q*(*w*) can be recast in terms of body length (*l*) using a suitable length-weight relationship such as *w* = *φl* ^*β*^, where *φ* and *β* are positive parameters. Eq (4) then becomes:

$$\begin{array}{ccc} E_{f_{y}}\left[C(l)\right] & = & \int_{l_{r}}\;\frac{q(l)}{l}\;f_{y}\left(l\right)\;dl\\ & = & \frac{k}{l_{\infty }^{\rho }\;A\;\left(1-d\right)}\int_{l_{r}}\;l^{\rho }\;f_{y}\left(l\right)\;dl\\ & = & \frac{k}{l_{\infty }^{\rho }\;A\;\left(1-d\right)}\;E_{f_{y}}\left[\;l^{\rho }\;\right]\;, \end{array}$$ (5)

where *ρ* = *β*(*d* − 1), *l*_∞_ is the asymptotic body length, *l*_0_ is the length-at-age zero, *l*_*r*_ ≔ [*l*_0_, *l*_∞_], and *f*_*y*_(*l*) is the pdf of lengths in the population at time t* in year *y*.

For brevity, we will refer to the expected population consumption to biomass ratio $\left(E_{f_{y}}[C]\right)$ at time t* in year *y*, as *C*_*y*_ hereafter. It is apparent that the estimation of *C*_*y*_ hinges on the estimation of the expected value of *l* ^*ρ*^. The modelled value of *C*_*y*_ represents the expected consumption to biomass ratio and it has units of *time* ^−1^. When *k* has units of *year* ^−1^, Eq (5) represents the number of times a population consumes its own weight over the year after t*. This expected consumption over the next year gets continuously updated as the expected value of the inverse of body length changes. To implement the model described in Eq (5), it is necessary to have estimates of individual growth parameters (*k* and *l*_∞_), assimilation rate (*A*) and the expected *l* ^*ρ*^ (*E*_*f*_*y*__[*l* ^*ρ*^]). *A* can be estimated from the daily ration, in an analogous way to that used by Pauly [2] to estimate parameters defining conversion efficiency. The estimation of *C*_*y*_ depends in part on what data are available to estimate the expected *l* ^*ρ*^. We explore this in the next section.

If an unbiased sample of lengths is available in a population, *E*_*f*_*y*__[*l* ^*ρ*^] can be easily computed as the weighted average *l* ^*ρ*^. However, an unbiased sample of lengths in the population is usually not available in fisheries, because the length samples have been subjected to size and/or age-based fishing selectivity. In such cases, an estimator of *E*_*f*_*y*__[*l* ^*ρ*^] can be obtained by modelling the pdf of lengths *f*_*y*_(*l*) in the population at the time t* in the year *y*. Let us assume that we have some suitable functional form for *f*_*y*_(*l*) where:

$$f_{y}\left(l\right)=\sum_{a}\;P_{y}\left(a\right)P_{y}\left(l|a\right)\;,$$ (6)

and *P*_*y*_(*a*), the probability mass function (pmf) of the age *a* of a randomly chosen fish. *P*_*y*_(*l*∣*a*) is the pdf of length *l* given age *a* in the population of fish in the year *y*. *P*_*y*_(*a*) can be interpreted as the relative abundance of a cohort of the age *a* in the population. It is determined by the magnitude of the recruitment and mortality rates experienced by a cohort up to the moment of observation.

Accordingly, the expected function of length in the population is defined by:

$$E_{f_{y}}\left(l^{\rho }\right)=\int_{l}l^{\rho }\sum_{a}\;P_{y}\left(a\right)\;P_{y}\left(l|a\right)\;dl=\sum_{a}\;P_{y}\left(a\right)\;\;E\;\left[l\;{\left(a\right)}^{\rho }\right]\;.$$ (7)

*P*_*y*_(*a*) can be obtained from population model output and $E\;[l\;{\left(a\right)}^{\rho }]$ can be modelled from the growth parameters by assuming that a population is a mixture of overlapping cohorts each one represented by a Gaussian distribution of the length-at-age [18]. In such case, $E[l{\left(a\right)}^{\rho }]$ can be easily described by the general VBGF as $E[l{\left(a\right)}^{\rho }]=l_{\infty }^{\rho }{\left[(1-\left(1-l_{0}/l_{\infty }\right)e^{-ka})\right]}^{\rho }$ [19]. Thus, *E*_*f*_*y*__[*l* ^*ρ*^] is defined by:

$$E_{f_{y}}\left[l^{\rho }\right]=l_{\infty }^{\rho }\;\sum_{a}\;\left[{\left[(1-\left(1-l_{0}/l_{\infty }\right)e^{-ka})\right]}^{\rho }\;P_{y}\left(a\right)\right]\;.$$ (8)

Thus, a model to estimate *C*_*y*_ in a population can be written by combining Eqs (5) and (8) as follows:

$$C_{y}\left(a\right)=\frac{k}{A(1-d)}\left[\sum_{a}\;{\left[(1-\left(1-l_{0}/l_{\infty }\right)e^{-ka})\right]}^{\rho }\;P_{y}\left(a\right)\right]\;.$$ (9)

Note that this model depends on age because length was replaced by age when using the growth parameters. This model also allows us to compute *C*_*y*_ for different population stages (e.g. juveniles, adults) by evaluating different age strata. The model in Eq (9) can be implemented if the von Bertalanffy parameters and *P*_*y*_(*a*) are known. The latter can be obtained from age-structured stock assessment model outputs. Note that in such populations where *t*_0_ is available instead of *l*_0_, the following mathematical equivalence should be applied: *l*_0_ = *l*_∞_[1 − exp(*kt*_0_)].

Here, the assumption of a stable age-distribution is a special case. In this case the age structure is proportional to the survival function [20], thus, *P*_*y*_(*a*) = *e* ^−*Ma*^/∑_*a*_ *e* ^−*Ma*^.

## Simulation

In this section we evaluate the differences, in terms of deviation between *C*_*y*_ estimates from the model in Eq 9 and the model proposed by Aydin [3], as follows:

$$C_{y}=\left[\frac{3k}{A}\right]\times \left[\frac{1}{Z_{y}}-\frac{2e^{\left(kt_{0}\right)}}{Z_{y}+k}+\frac{e^{\left(2kt_{0}\right)}}{Z_{y}+2k}\right]\times {\left[\frac{1}{Z_{y}}-\frac{3e^{\left(kt_{0}\right)}}{Z_{y}+k}+\frac{3e^{\left(2kt_{0}\right)}}{Z_{y}+2k}-\frac{e^{\left(3kt_{0}\right)}}{Z_{y}+3k}\right]}^{-1}\;.$$ (10)

Here *Z*_*y*_ is total mortality in the year *y*, while the other parameters are defined above. In order to compare Aydin’s model and the model proposed in Eq (9), *t*_0_ in Aydin’s model was replaced by *l*_0_ using the relationship between *t*_0_ and *l*_0_ given above.

Twenty seven harvested populations of different lifespans were simulated. Life history parameters defining these populations were obtained from the functional trade-off between life history parameters reported for fish. Firstly, populations with lifespans (*a*_*max*_) from 4 to 30 years were generated. Then for each population, the natural mortality rate (*M*) was calculated as function of *a*_*max*_ by *M* = 4.22/*a*_*max*_ [21]. The growth coefficient *k* was calculated as *k* = (2/3)*M* [22] and *t*_0_ was assumed to be proportional to the lifespan such as *t*_0_ = −0.05 *a*_*max*_. Values of *t*_0_ were transformed to *l*_0_ using the mathematical equivalence between these two parameters. In addition, we assume a knife-edge selectivity to be proportional to a third of the lifespan. This means that a fixed fishing mortality rate is inflicted to all age groups above *S*. Fishing mortalities (*F*) between 0 and 1.2 (year^−1^) were evaluated in each population assessed. In order to match the assumptions in Aydin’s model, a first simulation was set to have constant recruitment, *d* = 2/3, *β* = 3.

A second simulation was implemented to assess the effect of recruitment variability on estimates of *C*_*y*_. Following the procedure described above, three populations with *a*_*max*_ of 5, 15 and 25 years were chosen to represent fish with different life history strategies. Fishing mortality was assumed time-invariant and equal to natural mortality *F*_*y*_ = *M*, thus to represent a fully exploited population [23]. Three shapes for recruitment variability in each of the population selected were implemented. The first shape was a declining recruitment with a decreasing rate of 5% per year such as *R*(*y* + 1) = 0.95 *R*(*y*). Likewise, a increasing recruitment was simulated such as *R*(*y* + 1) = 1.05 *R*(*y*). Finally, a recruitment coming from a uniform random distribution in the range [0.8–12] was simulated.

The relative deviation between the model presented here and Aydin’s model was computed as follows:

$$deviation=\frac{C_{y}-C_{aydin}}{C_{y}}\;,$$ (11)

where *C*_*y*_ is the model proposed here in Eq (9) and *C*_*aydin*_ is the model in Eq (10).

## Applications

To illustrate the method, we applied the model in Eq (9) to two fish species using data from southern Chile (41°28’—57°00’S). The species considered were pink cusk-eel (*Genypterus blacodes*) and southern hake (*Merluccius australis*). These species are intensely fished in the austral zone of Chile by a multiespecies demersal fishery consisting of industrial vessels operating trawls and longlines. Pink cusk-eel population is divided into two stocks: Northern zone (41°28’- 47°00’S) and Southern zone (47°00’- 57°00’S).

Parameters in Eq (9) were obtained separately from other sources of data. These independent parameter estimates were then plugged into Eq (9) to compute a time series of *C*_*y*_ estimates in each stock described above. Estimates from Eq (9) were compared with estimates from the Aydin’s model in Eq 10. In most of fish species, *C*_*y*_ is only available from empirical or indirect models. Here we compare our results with empirical equation in Palomares and Pauly [4] in the case of southern hake. For eel-shape fish such as pink cusk-eel, we used the empirical equation described by Pauly et al. [24].

The population value of *C*_*y*_ for pink cusk-eel was evaluated for 1978 to 2004 for individuals between 0 and 16 years old, and for southern hake covering individuals between 0 and 24 years old. The estimated proportions at age by year (*P*_*y*_(*a*)) and total mortality *Z*_*y*_ and their variances, were taken from the regular stock assessment programs carried out under contract with the Chilean government [25] for pink cusk-eel, [26] for southern hake). The VBGF parameter *k* and *l*_0_ their variance were taken from [27] for southern hake and from [28] for pink cusk-eel. For the purposes of comparing the model proposed here with the model in Aydin [3] we assumed *d* = 2/3 and *β* = 3 thus *ρ* = −1. The parameters (*A*) were estimated from the daily ration *R*_*d*_. Following Ivlev [12] the food growth conversion efficiency (*K*) was estimated as the growth increment per food ingested *K* = (*dw*/*dt*)/*R*_*d*_, and then *A* was estimated from Temming’s model [15] (*K* = *A*[1 − (*w*/*w*_∞_)^(1 − *d*)^]). Daily rations of 7.12 and 5.72 *g* × *days* ^−1^ for pink cusk-eel and southern hake, respectively, were re-estimated from the information provided by Pool et al. [29]. Details of the parameters used in the application are in Table 1.

**Table 1:** Parameters used to illustrate the application of the estimator for *C*_*y*_ in Eq (9). *k*, *l*_∞_ and *l*_0_ and *d* are parameters of the von Bertalanffy growth function taken from [28] for pink cusk-eel (both stocks) and from [27] for southern hake. *β* is the length-at-weight scaling parameter, *A* is assimilation rate estimated from daily rations and *P*_*y*_(*a*) is the proportion of individuals at age *a* in time *y* taken from stock assessment outputs.

Parameters	Pink cusk-eel (north stock)	Pink cusk eel (south stock)	Southern hake
*k*(1/*year*)	0.186	0.147	0.080
*l*_∞_(*cm*)	111.43	123.45	121.00
*l*_0_(*cm*)	17.39	28.71	13.33
*β*	3	3	3
*d*	2/3	2/3	2/3
*A*	0.769	0.769	0.593
Years for *P*_*y*_(*a*)	1978–2004	1978–2004	1978–2004

A sampling distribution of $\hat{C_{y}}$ was computed by drawing estimates of each parameter in Eq (9) as follows: $\left\{\hat{k},\hat{l_{0}}\right\}$ were resampled from a bivariate normal distribution considering the asymptotic distribution of maximum likelihood estimates. Uncertainty in ${\hat{P}}_{y}\left(a\right)$ and ${\hat{Z}}_{y}$ was taken from the stock assessment outputs. Due to a lack of available information on the variance of the parameter *A*, we assumed it to have no error. Ninety-five percent confidence intervals (CIs) for $\hat{C_{y}}$ were obtained by the percentile method [30] based on 5000 samples.

## Results

### Results of simulations

**Fig 1:**
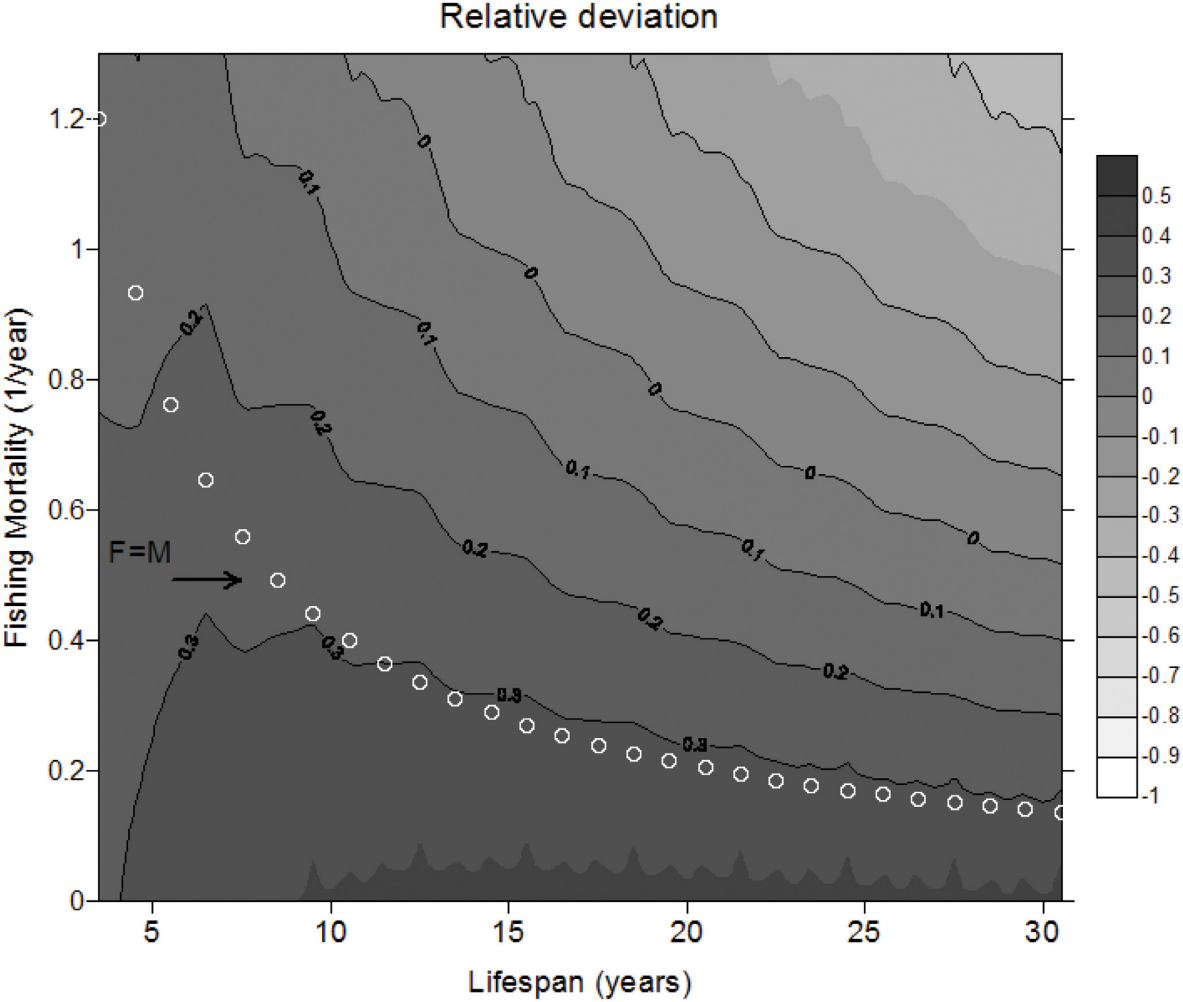
Relative deviation (Eq 11) between method presented here and the model in Aydin [3] across fishing mortality rate and lifespan. White circles represent the deviation for each lifespan for fully exploited populations (when *F* = *M*).

Fig 1 shows the relative deviation between the model presented here in Eq (9) and the model proposed in Aydin [3] in populations with different lifespans and across fishing mortalities. For a given fishing mortality, relative deviation decreases with lifespan. Likewise, for a given lifespan, relative deviation decreased with fishing mortality. Where large fishing mortalities are applied to relative long lifespan, the deviation became negative, meaning that estimates from the model presented here are smaller than those from Aydin’s model. In cases where *F* = *M*, deviation increases with the lifespan, from 0.1 to 0.3 in lifespan between 4 and 12 years. In population with lifespan older than 12 years, the deviation is relatively constant around 0.3 (Fig 1).

**Fig 2:**
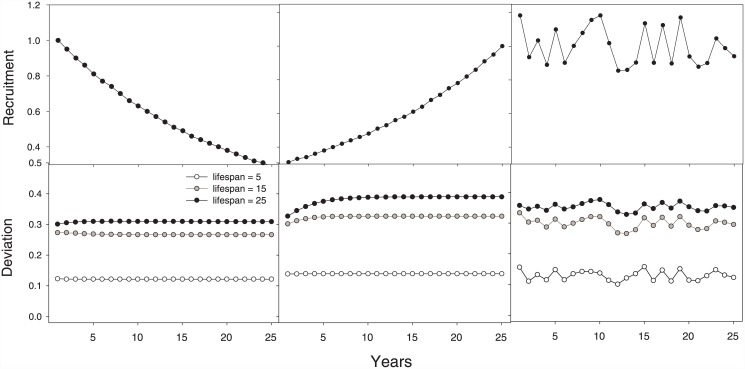
Relative deviation (Eq (11) between the method presented here and the model of Aydin [3] with variability in recruitment. The upper plots show the shape of recruitment variability across years and lower plots show the relative deviation for the three populations with different lifespans.

When variable recruitment was simulated across time (Fig 2), a similar behaviour is found between the lifespan and deviation reported in Fig 1. For a given time series of simulated recruitments, the deviation was larger in older populations (Fig 2). With declining recruitments, the deviation was almost constant across time for the three population types analysed. With increasing recruitment, the deviation slightly increases the first year to become constant for the last years of simulation. In addition, when recruitment has a random behaviour across time, the bias takes a similar pattern to recruitment across time in the three stocks analysed (Fig 2).

### Results of applications

**Fig 3:**
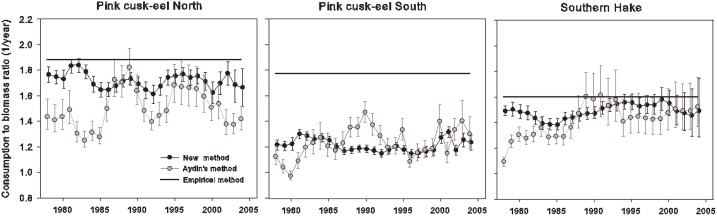
Estimates of *C*_*y*_ for the method proposed here, the model in Aydin [3] and the empirical equations for three fish populations off southern Chile. Vertical lines indicate the 95% confidence interval.

For the three stocks analysed, estimates of *C*_*y*_ by the model proposed here show less variation across time and narrower confidence intervals than estimates from Aydin’s model. In addition, mean estimates for the model presented here were always lower than predicted by the empirical equations applied to each evaluated stock (Fig 3). For the pink cusk-eel northern population, an average *C*_*y*_ of 1.72 was estimated with the model proposed here, compared to an average *C*_*y*_ of 1.50 estimated with Aydin’s model. For this stock, the empirical equation predicted a *C* value of 1.9. In the case of the pink cusk-eel southern population, the average estimated with the new model was 1.21 in comparison with 1.23 estimated with Aydin’s model. For this stock, the empirical equation predicted *C* to be 1.8. Finally, in the case of southern hake, average *C*_*y*_ estimated with the new model proposed here yielded 1.49 in comparison with 1.42 estimated with Aydin’s model. The empirical equation yielded an estimate of 1.6 for southern hake.

## Discussion

We have presented here a new model to estimate consumption that performs better than existing methods both on theoretical and empirical grounds, because it allows estimation of population consumption for populations that may or may not be in equilibrium. Aydin [3] improved over the model presented by Pauly [2] by allowing all parameters related to food consumption to be interpreted in biological terms. However, the model in Aydin [3] integrates consumption and biomass of a single recruit over its lifetime and therefore it is strictly valid as an estimate of current population consumption of stationary populations only. The cross-sectional estimate of *C*_*y*_ introduced here is directly applicable to populations that might be varying both in size and age-structure. In addition, the per-recruit model of Aydin for estimating *C*_*y*_ assumed that *Z*_*y*_ affected ages from 0+ equally. This is only applicable in populations subject to fishery removals were all ages have the same probability of being caught and *Z*_*y*_ is constant across years. In populations were *Z* varies across time, the population structure in one particular year will be a function of the sum of *Z*’s in prior years. Thus, per-recruit models are only likely to be realistic for estimating consumption to biomass ratio from cohorts with stable age-distribution which are not affected by fishing mortality. Lastly, analytical solutions to longitudinal models can only be obtained if a specialised form of the VBGF is used. We modelled consumption and biomass simultaneously, and this enables us to compute an instantaneous estimator for *C*_*y*_. This in turn allows us to propose a general model for *C*_*y*_ based on the generalised VBGF. It also allows us to relax the assumption of stable age-distribution, and to establish an explicit connection between *C*_*y*_ and body size. In this case, *P*_*y*_(*a*) contains the sum of mortalities in prior years. Our model allows us to explore the implications of the value of *C*_*y*_ for fishing exploitation in a natural way.

Although our model can in principle be applied to populations with stable age-distributions, the resulting estimator will still be different to that presented in Aydin [3] (see deviation in Fig 1 in cases with *F* = 0). These differences arise because these two models are actually estimating different quantities. Aydin’s estimator represents the consumption to biomass ratio of a cohort during its entire lifespan whereas our estimator represents consumption to biomass ratio for the entire population at one point in time. However, in the case of a similar ecological process, namely the production to biomass ratio, it has been demonstrated that cohort and population estimators compute different estimates [20]. Several authors (e.g. [31]) have suggested that the whole idea of cohort values for ratios such as the ratio of production to biomass should be abandoned when population estimators are available. Because of the mathematical and theoretical similarities between production and consumption, we believe the differences between cohort and population estimators found in production to biomass ratio can be extended to consumption. This underlying difference between Aydin’s model and the framework presented here explains the deviation showed in Fig 1 in cases where *F* = 0. This deviation also increases with lifespan because Aydin’s model relies on the assumption that fishing mortality is age-invariant so that fishing affects all recruited ages from age 0+ equally. In populations with a long lifespan, full recruitment to fishing gear may also occur at relatively older ages. This means that in older populations there will be more younger age groups that are only affected by natural mortality, while Aydin’s model assumes those ages are also affected by fishing mortality. Our model does account for late age of entry to the fishery so for populations with longer lifespans we observe larger relative deviation between the model proposed here and the model in Aydin [3]. In addition, the simulations in Fig 1 showed that in fully exploited populations (*F* = *M*) with lifespans of more than 10 years, our model gives estimates 30% higher than those from Aydin’s model. Likewise, in populations with shorter lifespans (< 10 years) this expected deviation between methods is about 20%. For heavily exploited populations (*F* > *M*) the deviation between methods for estimating *C*_*y*_ was smaller as lifespan decreased.

The models developed in this paper give a cross-sectional estimator of *C*_*y*_ in fish populations. These estimates can be interpreted as the potential consumption to biomass ratio of a fish population at a particular point in time. However, if a time series for *P*_*y*_(*a*) is available, the dynamic behaviour in *C*_*y*_ can also be determined by ordering estimates of this quantity at different points in time. Assuming that the VBGF for the species is time-invariant, the dynamic behaviour of *C*_*y*_ will depend on changes in *P*_*y*_(*a*) across time. As we have shown, *P*_*y*_(*a*) is determined by the relationship between recruitment and age-dependent mortality. In exploited fish populations, in which recruitment and fishing are concentrated in a short period of time, *P*_*y*_(*a*) will change once a year and its variation will be dependent on the relative magnitude of recruitment and age-dependent mortality changes between years. In cases where fishing is continuous between recruitment events, it is probably best to estimate *P*_*y*_(*a*) in the middle of the fishing season, as is the practice with annual stock assessments.

In the original formulation of the VBGF [32], the anabolism scaling parameter *d* is set at 2/3; the resulting model is known as the specialised VBFG. However, Essington et al. [5] compiled estimates for *d*, and concluded that its value differs from 2/3 for fish species. If *d* is allowed to take values other than 2/3, we have the generalised VBGF. However, parameters for the generalised VBGF cannot be taken directly from published sources, because they refer almost exclusively to the specialised VBGF. This is probably due to the impossibility of obtaining estimates for *d* solely from size-at-age data [33]. In order to estimate the other parameters of the generalised VBGF, *d* has to be fixed to a priori defined value. Pauly [33] proposed that for fish the slope of the linear relationship between gill area and body mass (0.789) can be used as an approximation for *d*, and Wiff and Roa-Ureta [13] concluded that this assumption was adequate for modelling consumption in fish. Such increase for the *d* value will cause estimates of *C*_*y*_ to be scaled up, and thus increasing the relative deviation between the model proposed here and the model proposed in Aydin [3].

A comprehensive understanding of food consumption by fish is difficult because it depends on a great number of internal (physiological) and external (environmental) factors [34]. Consumption rates in wild fish appear to be self-regulated, and ultimately determined by factors affecting metabolic rates [13, 35, 36] Temperature is one of the most important environmental variables affecting nearly all biological rates [37], and it has been identified as the most important factor shaping patterns of consumption (see [38]). For example, the empirical equation of Palomares and Pauly [4] and Pauly et al. [24] relates consumption to biomass ratio to habitat temperature. Although in the method develop here environmental factors are not explicitly incorporated, they can be considered as implicitly included because growth parameters and age structure can vary over time. Although the general framework of the VBGF [32] does not explicitly incorporate time-dependance in factors such as ambient temperature or food availability [39], it can be modified to do so by modelling the seasonal growth [40].

Population consumption to biomass ratio is one of the key quantities required to implement multiespecies models such as ECOPATH or ATLANTIS. In fished populations, ecosystem models are usually implemented with estimates of this ratio based on cohort per-recruit of empirical models. However, multiespecies models are based on the assumption that food consumption and other processes are observed at population levels or at stages of it (e.g. juveniles and adults life stages). Thus, multispecies models implemented using cohort per-recruit estimators of *C*_*y*_ will incorporates biases in model outputs. The size of such bias will depend on the deviation between *C*_*y*_ estimates using population vs cohorts per-recruit models. We have shown here that this deviation will depend mostly on the fishing mortality and life history parameters. Such bias may be of importance in ecosystem models because fished species are usually also a prey item of other fish species in higher tropic levels, thus, propagating this bias across the food web. Therefore, a population model for *C*_*y*_ such the one developed here, constitutes a promising method to introduce more realism in ecosystem models relaxing the assumptions of per-recruit and stable age-distribution when modelling *C*_*y*_.

## Supporting Information

S1 Dataset Consumption to biomass ratio data for Pink cusk-eel and Southern hake. (XLSX)
